# One-Hot Vector Hybrid Associative Classifier for Medical Data Classification

**DOI:** 10.1371/journal.pone.0095715

**Published:** 2014-04-21

**Authors:** Abril Valeria Uriarte-Arcia, Itzamá López-Yáñez, Cornelio Yáñez-Márquez

**Affiliations:** 1 Neural Networks and Unconventional Computating Lab/Alpha-Beta Group, Centro de Investigación en Computación, Instituto Politécnico Nacional, Ciudad de México, Distrito Federal, México; 2 Intelligent Computing Lab/Alpha-Beta Group, Centro de Innovación y Desarrollo Tecnológico en Cómputo, Instituto Politécnico Nacional, Ciudad de México, Distrito Federal, México; University of Westminster, United Kingdom

## Abstract

Pattern recognition and classification are two of the key topics in computer science. In this paper a novel method for the task of pattern classification is presented. The proposed method combines a hybrid associative classifier (Clasificador Híbrido Asociativo con Traslación, *CHAT*, in Spanish), a coding technique for output patterns called one-hot vector and majority voting during the classification step. The method is termed as CHAT One-Hot Majority (CHAT-OHM). The performance of the method is validated by comparing the accuracy of CHAT-OHM with other well-known classification algorithms. During the experimental phase, the classifier was applied to four datasets related to the medical field. The results also show that the proposed method outperforms the original CHAT classification accuracy.

## Introduction

Recognizing objects is an automatic routine task for humans and there is a myriad of problems involving pattern recognition. Simulating the human capacity for objects recognition has been a very important topic for computer sciences. For several decade, various approaches have been developed, which can be implemented on computers, to simulate the human ability to recognize objects. One of such approaches is the associative approach, whose main purpose is to correctly retrieve complete patterns from input patterns.

The first known model of associative memories is the Lernmatrix, developed in 1961 by Karl Steinbuch [1]. Some years later, an optical device capable of behaving as an associative memory was created by Buneman and Longuet-Higgins. [2]. In 1972, the work of Anderson [3], Kohonen [4], and to some extent Nakano [5], led to the model that is now known by the generic name of Linear Associator. In this same year Shun-Ichi Amari, published a theoretical work about self-organizing nets of threshold elements [6]. The work of Amari represents an essential background to one of the most important associative models: the Hopfield memory [7]. In the late 1980’s, Kosko [8] developed a bidirectional associative memory from two Hopfield memories. The morphological associative memories were introduced by Ritter *et al*. in 1998 [9], which represented a qualitative leap for associative models. These models incorporated concepts from mathematical morphology, which give them several advantages over the known models. Associative models have been widely and successfully used in different applications such as: pollutant concentration prediction [10], pattern classification [11], images processing [12], [13], among others.

In this paper, a method that combines a hybrid associative classifier, a coding technique for output patterns and majority voting, is presented. The rest of this paper is organized as follows. Section 2 describes all the materials and methods needed to develop our proposal. Section 3 describes how the experimental phase was conducted and discusses the results. Some conclusions are presented in Section 4 and finally the Acknowledge and References are included.

## Materials and Methods

### Associative Memories

An associative memory **M** is a system that relates input patterns and output patterns as follows [14]:
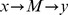



with x and y being the input and output patterns vectors. Each input vector form an association with its corresponding output vector. An associative memory is represented by a matrix whose 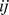-th component is 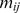. For each *k* integer and positive, the corresponding association will be denoted as: 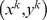. The matrix **M** is generated from a finite set of previously known associations, called the fundamental set. If *μ* is an index, the fundamental set is represented as: 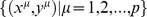, where 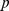 is the cardinality of the fundamental set. The patterns that form the fundamental set are called fundamental patterns. If it holds that 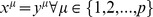, **M** is autoassociative, otherwise it is heteroassociative. If we consider the fundamental set of patterns 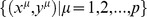 where 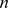 and 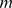 are the dimensions of the input patterns and output patterns, respectively, it is said that 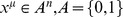 and 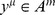. Then the *j*-th component of an input pattern 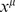 is 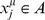. Analogously, the *j*-th component of an output pattern 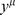 is represented as 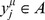. Therefore, the fundamental input and output patterns are represented as follows:



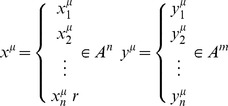



A distorted version of a pattern 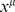 to be recalled will be denoted as 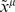. An unknown input pattern to be recalled will be denoted as 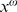. If when an unknown input pattern 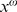 is fed to an associative memory **M**, and it happens that the output corresponds exactly to the associated pattern, 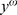 it is said that recalling is correct.

### Lernmatrix

The Lernmatrix is a heteroassociative memory that can function as a binary pattern classifier if the output patterns are properly selected [14]. It is an input and output system that accepts a binary input pattern 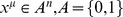 and produce as an output the class 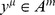. For a class 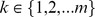, where *m* is the number of classes in the fundamental set, the class is coded according to the following expression: 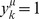 and 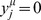 for 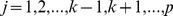. The Lernmatrix is represented by a matrix **M**. At the beginning of the learning phase, each component 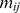 of **M** is set to zero and then it is updated according to rule 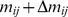, where:
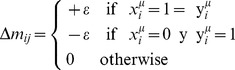
Where 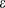 is any positive constant that was previously chosen.

The recovery phase consists of finding the class vector for a given vector 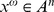. Finding the class means to obtain the coordinates of the vector 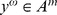 that corresponds to the pattern 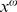. The *i*-th component 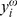 of the class vector 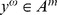 is obtained according to the following expression:
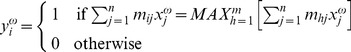



### Linear Associator

Let 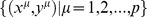 be the fundamental set with [15]:
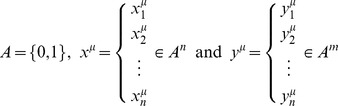



The learning phase consists of two steps:

1. For each of the 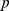 associations 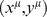 find the matrix 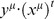 of dimensions 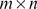

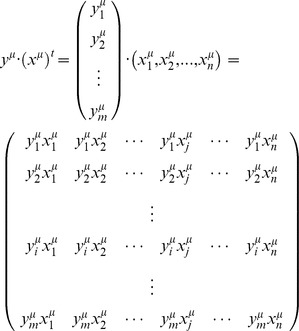

1. Sum the 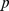 matrices to obtain the memory 
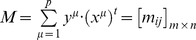
 where the *ij*-th component of **M** can be expressed as follow: 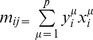.

The recovering phase consists of presenting an input pattern 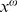 to the memory **M** and performing the following operation:
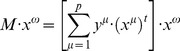



### CHAT

The CHAT is a hybrid associative classifier developed by Santiago-Montero [16], which is based on two associative memories: the *Lernmatrix* and the *Linear Associator*. This classifier overcomes some limitations that these two memories presented, by ingeniously combining the learning and recovery phases of both models. The first proposed model was called CHA, which combined the learning phase of the Linear Associator and the recovering phase of the Lernmatrix, but sometimes this model fails to perform a correct classification. To overcome this limitation, a new version was proposed, by adding a new step to the model: translation of coordinates axes. This new version was named CHAT. With this axes translation, the new origin is located at the centroid of the input vectors patterns.

Definition 3.1: Let 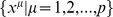 be a set of fundamental input patterns, and let 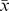 be the mean vector of them, where:
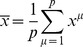



Definition 3.2: Let 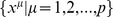 be a set of fundamental input patterns and 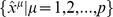 a new set of translated patterns generated using the following expression:
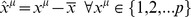



### CHAT Algorithm

1. Let 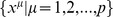 be a set of *n*-dimensional fundamental input patterns with real values in its components, which are grouped into *m* classes.
2. To each of the fundamental input patterns belonging to class *k,* an output vector of size *m* is assigned. This vector consists of zeros, except for the *k*-th component, whose value is set to 1.
3. Calculate the mean vector of the set of input patterns according to definition 3.1.
4. The mean vector is taken as the new origin of the coordinate axes.
5. Translate the patterns of the input set according to definition 3.2.
6. Apply the learning phase, which is the same as the learning phase of the Linear Associator, to the translated set obtained in the previous step.
7. Translate the patterns that have to be classified using the definition 3.2.
8. Apply the recovering phase, which is the same as the recovering phase of the Lernmatrix, to the translated set obtained in the previous step.

### CHAT-OHM

In this section the description of the proposed method is presented. This proposal is part of the results achieved by several members of the Neural Networks and Unconventional Computing Laboratory of the *Centro de Investigación en Computación, Instituto Politécnico Nacional*, in an attempt to improve the performance of the CHAT model [16]. This joint effort resulted in a number of methods that implemented some variations on the CHAT, being the proposed method one of them.

### One-hot Vectors

One-hot vector is a coding technique for output patterns, which will be used in the proposed method instead of the original coding technique presented in the step 2 of the CHAT algorithm that was described in the previous section.

Definition 3.3: Let 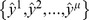 be a set of translated fundamental output patterns of size *p*. The *i*-th component of each translated fundamental output pattern is coded according to the following expression:
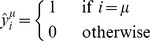



### Majority Voting

The classification phase consists of finding the output vector 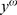 to which an unknown input pattern 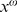 belongs. Majority voting is a procedure used during the classification phase to perform this task.

Definition 3.4: Let 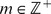 be the number of different classes in the fundamental input set. Let 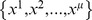 be a set of fundamental input patterns where 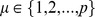. For a class 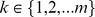, a masking vector 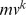 of size 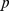 is coded according to the following expression:
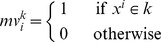



Let **M** be a matrix generated during the learning phase of the CHAT-OHM and 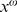 an unknown *n*-dimensional input pattern to be classified. The recover pattern 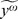 is determined as follow:


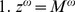

1. For each class 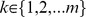, a counting vector 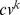 is obtained applying an “and” operator between 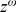 and 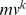:

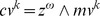

1. Finally 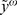 is obtained using the following expression:

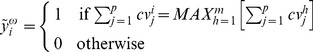



### CHAT-OHM Algorithm

1. Let 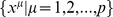 be a set of *n*-dimensional fundamental input patterns with real values in its components, which are grouped into *m* classes.
2. To each of the fundamental input patterns an output vector of size *p* is assigned. This vector is coded according to the definition 3.3.
3. Calculate the mean vector of the set of input patterns according to definition 3.1.
4. The mean vector is taken as the new origin of the coordinate axes.
5. Translate the patterns of the input set according to definition 3.2.
6. Apply the learning phase, which is the same that as learning phase of the Linear Associator, to the translated set obtained in the previous step.
7. Translate the patterns that have to be classified using the definition 3.2.
8. Apply the recovering phase, which is the same as the recovering phase of the Lernmatrix, to the translated set obtained in the previous step. Because of the way in which the classes were coded, we will obtain an output vector 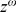 of size *p* and not the desire output class 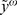 of size *m*. Our algorithm performs an extract step.
9. Perform the majority voting explained in the previous section.

### Data Sets

This section provides a brief description of the dataset used during the experimental phase. All the used datasets were taken from the University of California at Irvine Machine Learning Repository [17]. A summary of the main characteristics of the datasets is shown in Table 1.

**Table 1:** Characteristics of the datasets used in the experimetal phase.

Dataset	Instances	Attributes	Missing Values
**Breast Cancer**	683	9	Yes
**Haberman’s Survival**	306	3	No
**Liver Disorders**	345	6	No
**Hepatitis Disease**	155	19	Yes

### Haberman’s Survival Dataset

The dataset contains cases from a study conducted at the University of Chicago’s Billings Hospital on the survival of patients who had undergone surgery for breast cancer. The dataset contain 306 instances, which belong to two different classes; 255 instances belong to the first class (patients who survived 5 years or more) and 81 instances belong to the second class (patients who died within 5 years). The dataset has 4 attributes including the class attribute. The purpose of the dataset is to predict the survival status of patients that have undergone breast cancer surgery.

### Wisconsin Breast Cancer Dataset

This dataset was obtained from the University of Wisconsin Hospitals, Madison from Dr. William H. Wolberg. The dataset has information of clinical cases of breast cancer. The dataset contains 699 instances belonging to two classes, 458 instances belong to the first class (benign) and 241 belong to the second class (malignant). Each instance consists of 10 attributes, including the class attribute. The dataset has 16 pattern with one missing values. The instances with missing values were deleted from the original dataset and the resulting data set was used for the experimental phase.

### Liver Disorders Dataset

The Liver Disorders dataset was created by BUPA Medical Research Ltd. This dataset presents the results of a study of liver disorders that might arise from excessive alcohol consumption. It contains 345 instances belonging to two classes, 145 instances belong to the first class and 200 instances belong to the second class. Each instance consists of 7 attributes, including the class attribute.

### Hepatitis Disease Dataset

This dataset contains information of the clinical results of hepatitis patients. It contains 155 instances belonging to two classes, 32 instances belong to the first class (die) and 123 instances belong to the second class (alive). Each instance consists of 20 attributes, including the class attribute. This dataset has multiple missing values. Due to the small size of the dataset and the considerable number of missing values, these cannot be discarded. In this case the missing values were substituted by the class mode for categorical features and by the class mean for continuous values.

### Machine Learning Algorithms

This section provides a short description of the algorithms used during the experimental phase. All of these algorithms are implemented in the WEKA 3: Data Mining Software in Java [18]. Further details on the implementation of these algorithms can be found in the following reference [19].

### IB1

*IB1* is a basic nearest-neighbor instance-based learner that finds the training instance closest in Euclidean distance to the given test instance and predicts the same class as this training instance. If several instances qualify as the closest, the first one found is used [19].

### ConjunctiveRule

*ConjunctiveRule* learns a simple conjunctive rule learner that predicts either a numeric or a nominal class value. Uncovered test instances are assigned the default class value of the uncovered training instances. The information gain (nominal class) or variance reduction (numeric class) of each antecedent is computed, and rules are pruned using reduced-error pruning [19].

### RandomTree

Trees built by RandomTree test a given number of random features at each node, performing no pruning. The tree is constructed considering K randomly chosen attributes at each node. Also has an option to allow estimation of class probabilities based on a hold-out set [19].

### RandomForest

*RandomForest* constructs random forests by bagging ensembles of random trees. A random forest is a classifier consisting of a collection of tree-structured classifiers and each tree depends on the values of a random vector sampled independently and with the same distribution for all trees in the forest [20].

### BFTree

BFTree constructs a decision tree using a best-first expansion of nodes rather than depth-first expansion used by standard decision tree learners. Pre and post pruning option are available that are based on finding the best number of expansion to use via cross-validation on the training data. While fully grown trees are the same for best-first and depth-first algorithms, the pruning mechanism used by BFTree will yield a different pruned tree structure than that produced by depth-first methods [19].

### SMO

*SMO* implements John Platt’s sequential minimal optimization algorithm for training a support vector classifier, using kernel functions such as polynomial or Gaussian kernels. Missing values are replaced globally, nominal attributes are transformed into binary ones, and attributes are normalized by default. For further details of the implementation, see [21].

### AdaBoostM1

*AdaBoostM1* is a variation of boosting, method for combining multiple models seeking models that complement one another. This algorithm is constructed through the combination of various classifiers produced by repeatedly running T rounds a given “weak” learning algorithm on various distributions over the training data. Finally the booster combine the T “weak” hypotheses into a single final hypothesis [22].

### MultiBoostAB

MultiBoostAB combines boosting with a variant of wagging to prevent overfitting. Multiboosting is an extension of AdaBoost technique [23]. Wagging is a technique that allow variance reduction, while AdaBoost perform both variance and bias reduction. MultiBoost is achieved by wagging a set of sub-committees of classifiers, each sub-committee formed by AdaBoost. When forming decision committee using C4.5 as the base learning algorithm, MultiBoost is demonstrated to produce committees with lower error than AdaBoost.

### RBFNetwork

*RBFNetwork* implements a normalized Gaussian radial basis function network, deriving the centers and widths of hidden units using k-means and combining the outputs obtained from the hidden layer using logistic regression if the class is nominal and linear regression if it is numeric. The activations of the basis functions are normalized to sum to 1 before they are fed into the linear models [19].

### NaiveBayes

*NaiveBayes* implements the probabilistic Naïve Bayes classifier. The NaiveBayes algorithm is based on Bayes rule and assumes that the attributes are conditional independent given the class, and it posits that no hidden or latent attributes influence the prediction process [24].

### BayesNet

Bayesian networks are alternative ways of representing a conditional probability distribution by means of directed acyclic graphs (DAGs). In this model, each node represents a random variable and an arrow connecting a parent node with a child node indicates a relationship between them [25]. *BayesNet* learns Bayesian nets under two assumptions: nominal attributes (numeric ones are pre-discretized) and no missing values (any such values are replaced globally).

### NaiveBayesMultinomial

NaiveBayesMultinomial implements the multinomial Bayes’ classifier. A Naïve Bayes classifier is based on Bayes rule but this does not take into account the number of occurrence of an element. The Naïve Bayes Multinomial incorporates frequency to perform classification [19].

### ComplementNaiveBayes

ComplementNaiveBayes builds a Complement Naïve Bayes classifier as described by Rennie *et al* [26]. In this work, they proposed heuristic solutions to some problems presented by the Naïve Bayes classifiers. They proposed a solution for skewed data, more training examples for one class than another that causes that the classifier prefer one class over the other.

### DecisionTable

DecisionTable builds a simple decision table majority classifier, this table has two components: a set of features that are included in the table and a body consisting of labeled instances from the space defined by the features [27].

### LWL

LWL is a general algorithm for locally weighted learning. It assigns weights using an instance-based method and builds a classifier from the weighted instances. Different classifiers can be selected, but a good choice is Naïve Bayes for classification problems and linear regression for regression problems. Attribute normalization is turned on by default [19].

### DMNBtext

Another Naïve Bayes scheme for text classification is DMNBtext. This learns a multinomial Naïve Bayes classifier in a combined generative and discriminative fashion. DMNBText injects a discriminative element into parameter learning by considering the current classifier’s prediction for a training instance before updating frequency counts. When processing a given training instance, the counts are incremented by one minus the predicted probability for the instance’s class value. DMNBText allows users to specify how many iterations over the training data the algorithm will make, and whether word frequency information should be ignored, in which case, the method learns a standard Naïve Bayes model rather than a multinomial one [19].

### MultiScheme

MultiScheme selects the best classifier from a set of candidates using cross-validation of percentage accuracy or mean-squared error for classification and regression, respectively. The number of folds is a parameter. Performance on training data can be used instead [19].

### Vote

Vote provides a baseline method for combining classifiers. The default scheme is to average their probability estimates or numeric predictions, for classification and regression, respectively. Other combination schemes are available–for example, using majority voting for classification [19].

### VotedPerceptron

VotedPerceptron implements the voted perceptron algorithm. The solution vector found by the perceptron algorithm depends greatly on the order in which the instances are encountered. One way to make the algorithm more stable is to use all the weight vectors encountered during learning, not just the final one, letting them vote on a prediction. Each weight vector contributes a certain number of votes [19].

### Normalization

During the experiments performed over the original data, we observed that some of the datasets present large scale difference between features. To avoid the effect that an overly large variable can have over the classification performance, the datasets were normalized and the experiments were performed with the normalized datasets. Normalization can prevent some features from dominating just because they have large numeric values. Subtracting the mean and dividing by the standard deviation can be an appropriate normalization method for this situation [31]. The normalization was performed separately on each attribute. Normalization was calculated using the following expression:
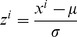
Where 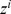 is the normalized value of 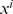, *μ* is the mean of the population and *σ* is the standard deviation of the population.

### Wilson’s Edition

One of the most popular filtering algorithms is the Wilson’s Edition [28]. The general idea of this method is to identify and remove noisy or atypical patterns, primarily those which exist in the overlap area between two or more classes. The process consists of applying the rule of the *k* nearest neighbor (usually *k* = 3) to estimate the corresponding class of each pattern in the dataset. Those patterns whose class does not correspond to the majority class of the *k*-nearest neighbors will be discarded [28].

### Algorithm Comparison

One of the objectives of this study is to perform a consistent comparison between the classification performance of our proposal and the classification performance of other well-known pattern classification algorithms. There are two aspects that need to be addressed: select a suitable test set and the method to compare the classification performance of each algorithm. To predict the performance of a classifier, we need to assess the success rate on a dataset that takes no part in the construction (training phase) of the classifier. When the data available is big, there is no problem in the selection of a suitable test set, just use a large training set and a large test set. But the question of predicting performance with limited data is still controversial. There are many techniques, of which cross-validation is the method of choice in most situations. Kohavi [29] compared cross-validation and bootstrap, the results show that bootstrap has low variance, but extremely large bias for some problems; as a consequence stratified 10-fold cross-validation is recommended. To perform the comparison of our proposal with other pattern classification algorithms, we used the 10-fold cross-validation approach.

### Classification Accuracy

For classification problems, the performance of a classifier can be measured in term of the success rate. The classifier predicts the class of each instance in the test set; if the class is correct, it is counted as a success. The success rate is the proportion of success over the whole set of test instances. In this paper, the accuracy of the classifiers is expressed as a percentage, and was computed according to the following expression:
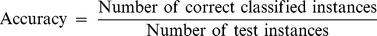



### Validation Method

According to [19] the standard way of predicting the classification accuracy of a learning technique is to use stratified 10-fold cross-validation. This method divides the dataset into 10 parts in which each class is represented in approximately the same proportion as in the full dataset. The classification algorithms will be executed 10 times, in each execution one different part will be used as the test set and the classification algorithm will be trained with the remaining nine parts. The success rate will be calculated for each execution. Finally, the 10 success rates are averaged to yield an overall success rate.

## Experiments and Discussion

In this section we present and discuss the results obtained during the experimental phase, throughout which four datasets were used to obtain the classification performance of each of the compared classification algorithms. The datasets used in this section were taken from the UCI Machine Learning Repository [17]. A brief summary of the datasets is presented in Table 1.

The performance achieved by the proposed method is compared with the performances of 19 well-known methods taken from the WEKA 3 Data Mining Software [18]. Further information about the used algorithms can be found in [19]. All experiments were conducted using a personal computer with an Intel Core i3-2100 Processor running Ubuntu 13.04 64-bits operating system with 4096 GB of RAM.

To ensure valid comparison of classification performance, the same conditions and validation schemes were applied in each experiment. Classification performance of each of the algorithms was calculated using stratified 10-fold cross-validation, with random re-ordering of the patterns before fold generation. In order to account for the random re-ordering of the patterns, the experiments for each classification algorithm, including our proposal, were executed 10 times using the stratified 10-fold cross-validation approach and the results averaged to obtain a final success rate for each algorithm. These results are used to compare the performance of our proposal and the other classification algorithms.

### Original Datasets

In this subsection we analyze the classification accuracy results of each one of the compared algorithms, when applied to the original four datasets that were selected for this study. Table 2 shows the classification accuracy achieved by the original CHAT model and by our proposal in the four datasets. It is worth noting that CHAT-OHM achieved the best classification accuracy for all the datasets. In some cases the improvement in the classification accuracy is quite significant, as in the cases of the Breast Cancer dataset and the Hepatitis Disease dataset, with an improvement of 31.9 percent and 16.77 percent, respectively. The improvement for the Liver Disorders dataset is 5.82 percent, which is still important. The Haberman’s Survival dataset is where we observed the least improvement with only 0.41 percent.

**Table 2:** Accuracy comparison with the original method (%) original data.

	Datasets			
Algorithm	Breast Cancer	Haberman’s Survival	Hepatitis Disease	Liver Disorders
**CHAT**	63.10	65.95	68.19	55.63
**CHAT-OHM**	95.00	66.36	84.96	61.45

Table 3 shows the classification accuracy achieved by our proposal and the 19 classification algorithms from WEKA, against which we will compare our method. For each dataset, the highest classification accuracy is emphasized with boldface.

**Table 3:** Clasification accuracy comparison (%) original data.

	Dataset			
Algorithm	Breast Cancer	Haberman’s Survival	Hepatitis Disease	Liver Disorders
**AdaBoostM1**	95.05	74.02	89.8	65.96
**BayesNet**	**97.34**	71.73	87.68	56.85
**BFTree**	94.88	72.33	88.98	66.44
**CHAT-OHM**	95.00	66.36	84.96	61.45
**ComplementNaiveBayes**	85.43	73.87	77.54	56.57
**ConjunctiveRule**	91.98	72.97	88.99	56.06
**DecisionTable**	95.69	71.90	88.40	59.11
**DMNBtext**	65.01	73.53	79.38	57.98
**IB1**	95.75	65.77	81.03	62.22
**LWL**	92.14	71.90	89.70	60.80
**MultiBoostAB**	94.73	73.28	89.49	65.29
**MultiScheme**	65.01	73.53	79.38	57.98
**NaiveBayes**	96.26	**74.80**	87.50	54.89
**NaiveBayesMultinomial**	90.32	73.74	78.00	56.96
**RandomForest**	96.47	67.94	**90.61**	**68.44**
**RandomTree**	94.74	64.48	85.32	64.10
**RBFNetwork**	96.36	73.75	85.78	65.06
**SMO**	96.87	73.33	88.83	57.98
**Vote**	65.01	73.53	79.38	57.98
**VotedPerceptron**	91.08	73.82	78.09	63.53

As we can observe in Table 3, the CHAT-OHM does not surpass all the other classification algorithms, still it exhibits a competitive classification accuracy. For the Breast Cancer dataset the CHAT-OHM achieved a performance of 95% (9th place), only 2.34% below the best performer, BayesNet. For the Liver Disorders dataset the best classifier was RandomForest, with a 68.44% of classification accuracy, while the CHAT-OHM reached the 9th place with a difference of performance of 6.99%. For the case of Haberman’s Survival dataset CHAT-OHM achieved a classification accuracy of 66.36% which leaves it in 18th place with 8.44% below the best classifier, NaiveBayes. The best performance for Hepatitis Disease dataset was achieved by RandomForest with 90.61% of classification accuracy, while the CHAT-OHM was positioned in the 13th place with a classification accuracy of 84.96%.

Notice, however, that despite not exhibiting the best performance for any given dataset, CHAT-OHM has a consistent behavior: the proposed method reached the 9th place for the Breast Cancer dataset and the Liver Disorders dataset, while being the 13th place for the Hepatitis Disease dataset and the 18th place for the Haberman’s Survival dataset. On the other hand, Bayes Net is the best classifier for the Breast Cancer dataset, while being the 17th place for the Liver Disorders dataset, the 16th place at the Haberman’s Survival dataset, and the 9th place for the Hepatitis Disease dataset. Another example of this inconsistent performance is that of the NaiveBayes algorithm: it is the 5th place for Brest Cancer dataset, the worst method for the Liver Disorders dataset, the best at Haberman’s Survival dataset, and the 10th method for Hepatitis Disease dataset.

### Normalized Datasets

While performing the experiments, we noticed that some attribute values are significantly larger than the values of the rest of the attributes. As recommended by [31] to avoid the impact of scale change, the dataset can be normalized. The justification usually given for this normalization is that it prevents certain features from dominating merely because they have large numerical values.

Table 4 shows the classification accuracy achieved by our proposal and the 19 classification algorithms from WEKA, when applied to normalized datasets. For each dataset, the highest classification accuracy is emphasized with boldface. In general, no significant variations were achieved with respect to the results of the datasets without normalization. In most cases the improvement is less than 2 percent, with only two clear exceptions: VotedPerceptron and DMNBtext, which significantly increased their classification accuracy. The former exhibits an improvement of 5.79% for the Breast Cancer dataset and 6.94% for the Hepatitis Disease dataset, while the latter shows an improvement of 24.99% for the Breast Cancer dataset, 6.19% for the Liver Disorders dataset and 9.61% for the Hepatitis Disease dataset.

**Table 4:** Clasification accuracy comparison (%) normalized data.

	Dataset			
Algorithm	Breast Cancer	Haberman’s Survival	Hepatitis Disease	Liver Disorders
**AdaBoostM1**	95.05	74.02	89.80	67.72
**BayesNet**	**97.34**	71.73	87.68	56.62
**BFTree**	94.80	72.43	88.92	67.15
**CHAT-OHM**	95.52	62.45	89.52	58.5
**ComplementNaiveBayes**	N/A	N/A	N/A	N/A
**ConjunctiveRule**	91.98	72.97	89.05	56.28
**DecisionTable**	95.69	71.90	88.46	58.83
**DMNBtext**	90.00	73.01	88.99	64.17
**IB1**	95.46	65.58	81.03	63.25
**LWL**	92.14	71.90	89.95	60.74
**MultiBoostAB**	94.73	73.28	89.49	64.29
**MultiScheme**	65.01	73.53	79.38	57.98
**NaiveBayes**	96.11	74.66	87.36	55.42
**NaiveBayesMultinomial**	N/A	N/A	N/A	N/A
**RandomForest**	96.33	67.81	**90.54**	**68.44**
**RandomTree**	94.79	64.96	83.70	62.75
**RBFNetwork**	96.36	73.75	85.78	64.81
**SMO**	96.88	73.53	88.70	57.90
**Vote**	65.01	73.53	79.38	57.98
**VotedPerceptron**	96.87	**75.09**	85.03	65.86

The performance of CHAT-OHM was not significantly affected by normalization, but for the Hepatitis Disease dataset the improvement of 4.56% changes its rank from the 13th place (Table 3) to the 4th place (Table 4).

The normalization method used in our experiments, produce both positive and negative normalized values. This situation did not allow us to perform the experiment with two classification algorithms from WEKA: ComplementNaiveBayes and NaiveBayesMultinomial, since these algorithms are unable to handle negative values.

### Outliers Treatment

During the testing phase, we also noticed the presence of some atypical pattern in the datasets. To verify the presence of outliers in the datasets, a method for detection and deletion of outliers called Wilson’s Edition was applied to the datasets [28]. Table 5 shows the amount of outliers found and deleted from the four datasets using this technique. The information presented by this table, shows that the Breast Cancer dataset presents only 3.22% of outliers while Haberman’s Survival dataset presents 36.45%. The fact that most of the classifiers work much better for the Breast Cancer dataset, may be justified by the almost absence of outliers in this dataset. The decision boundary between the classes appears to be better defined for the Breast Cancer dataset, thus the classification algorithms exhibit a higher classification accuracy than the one achieved with the other datasets, where the decision boundaries seem not so well defined.

**Table 5:** Number of outliers for dataset.

	Original Dataset			Outliers			
	Total Instances	Class 1 Instances	Class 2 Instances	Class 1 Outliers	Class 2 Outliers	Total Outliers	Outliers %
**Breast Cancer**	683	444	239	11	11	22	3.22
**Haberman’s Survival**	345	145	200	71	55	126	36.52
**Hepatitis Disease**	306	225	81	25	59	84	27.45
**Liver Disorders**	155	32	123	14	13	27	17.41

Table 6 shows the classification accuracy achieved by our proposal and the 19 classification algorithms from WEKA, when applied to datasets without outliers. For each dataset, the highest classification accuracy is emphasized with boldface. The removal of outliers leads to an improvement for all the classification algorithms presented in this work. For the Breast Cancer dataset 22 outliers were removed, which represent the 3.22% of the original dataset. The improvement in the classification accuracy for this dataset varies from 0.5% to 4.27%. The CHAT-OHM shows an improvement of 3.95% for the Breast Cancer dataset, which changes its position from the 9th place (Table 3) to the 4th place (Table 6), as mentioned before.

**Table 6:** Clasification accuracy comparison (%) data without outliers.

	Dataset			
Algorithm	Breast Cancer	Haberman’s Survival	Hepatitis Disease	Liver Disorders
**AdaBoostM1**	98.41	94.84	96.24	83.44
**BayesNet**	99.46	87.59	93.72	76.98
**BFTree**	97.84	93.39	96.40	84.43
**CHAT-OHM**	98.95	90.44	88.94	69.23
**ComplementNaiveBayes**	86.79	90.60	86.72	64.44
**ConjunctiveRule**	93.78	89.52	95.25	66.53
**DecisionTable**	97.16	88.32	94.31	77.73
**DMNBtext**	65.51	90.12	86.03	66.21
**IB1**	99.35	93.70	87.71	75.13
**LWL**	96.41	91.00	95.71	66.95
**MultiBoostAB**	97.25	92.41	96.32	80.11
**MultiScheme**	65.51	90.12	86.03	66.21
**NaiveBayes**	97.93	**95.25**	**97.63**	58.54
**NaiveBayesMultinomial**	92.24	90.93	87.72	65.44
**RandomForest**	98.55	93.79	96.10	**87.36**
**RandomTree**	97.75	92.85	93.65	81.98
**RBFNetwork**	98.46	93.26	96.62	79.03
**SMO**	**99.50**	91.24	92.63	66.21
**Vote**	65.51	90.12	86.03	66.21
**VotedPerceptron**	92.90	90.39	85.40	73.89

Table 7 shows the classification accuracy achieved by our proposal and the 19 classification algorithms from WEKA, when applied to normalized datasets without outliers. For the Liver Disorder dataset, increases in the classification accuracy can be observed when compared with the experiments performed on the original datasets. But if we compare the result of experiments with the datasets without outliers and the ones with the normalized datasets without outliers, the classification accuracy gets worse instead of better. In general, it seems that for this dataset, it is better not to use normalization and instead rely on the removal of outliers. On the other hand CHAT-OHM performed better with the normalized and outliers-free dataset. The original performance was 61.45%, the performance with the outliers-free dataset was 69.23% and the performance with the normalized outliers-free dataset was 74.13%; with this improvement the classifier changes its rank from the 9th place with the original dataset (Table 3) to the 4th place with the normalized outliers-free dataset (Table 7).

**Table 7:** Clasification accuracy comparison (%) normalized data without outliers.

	Dataset			
Algorithm	Breast Cancer	Haberman’s Survival	Hepatitis Disease	Liver Disorders
**AdaBoostM1**	98.41	94.84	96.24	75.58
**BayesNet**	99.47	87.59	93.72	66.80
**BFTree**	97.70	93.75	96.40	75.63
**CHAT-OHM**	97.69	90.53	89.18	74.13
**ComplementNaiveBayes**	N/A	N/A	N/A	N/A
**ConjunctiveRule**	93.78	89.52	96.33	61.41
**DecisionTable**	97.17	88.32	94.31	68.28
**DMNBtext**	94.49	90.12	93.02	69.28
**IB1**	99.35	93.70	87.71	69.19
**LWL**	96.41	91.00	95.71	63.85
**MultiBoostAB**	97.25	92.42	96.32	72.20
**MultiScheme**	65.51	90.12	86.03	62.10
**NaiveBayes**	97.79	**95.20**	**97.02**	56.85
**NaiveBayesMultinomial**	N/A	N/A	N/A	N/A
**RandomForest**	98.70	93.75	96.39	**77.26**
**RandomTree**	97.65	92.77	91.67	71.97
**RBFNetwork**	98.46	93.26	96.92	71.92
**SMO**	99.50	91.33	92.63	62.05
**Vote**	65.51	90.12	86.03	62.10
**VotedPerceptron**	**99.88**	93.63	86.97	72.10

### Improvement Analysis

From the results presented in Table 3, 4, 6, and 7, it is shown that there is no specific classification algorithm that exceed all the other algorithms in all the presented problems. This claim is supported by the No-Free-Lunch Theorems presented by Wolpert and Macready [30], which establish that for an algorithm, any performance gain in one kind of problem is offset by its performance loss in other kind of problems.

Table 8, 9, 10, and 11 show the percentage of improvement achieved by our proposal and the 19 classification algorithms from WEKA, when applied to normalized datasets, datasets without outliers, and normalized datasets without outliers, for each of the four datasets used.

**Table 8:** Comparison of classification Improvement (%) for Breast Cancer dataset.

	Breast Cancer		
Algorithm	Normalization	Without Outliers	Without Outliers Normalized
**AdaBoostM1**	0.00	3.36	3.36
**BayesNet**	0.00	2.12	2.13
**BFTree**	−0.08	2.96	2.82
**CHAT-OHM**	0.52	3.95	2.69
**ComplementNaiveBayes**	N/A	1.36	N/A
**ConjunctiveRule**	0.00	1.80	1.80
**DecisionTable**	0.00	1.47	1.48
**DMNBtext**	**24.99**	0.50	**29.48**
**IB1**	−0.29	3.60	3.60
**LWL**	0.00	**4.27**	4.27
**MultiBoostAB**	0.00	2.52	2.52
**MultiScheme**	0.00	0.50	0.50
**NaiveBayes**	−0.15	1.67	1.53
**NaiveBayesMultinomial**	N/A	1.92	N/A
**RandomForest**	−0.14	2.08	2.23
**RandomTree**	0.05	3.01	2.91
**RBFNetwork**	0.00	2.10	2.10
**SMO**	0.01	2.63	2.63
**Vote**	0.00	0.50	0.50
**VotedPerceptron**	5.79	1.82	8.8

**Table 9:** Comparison of classification improvement (%) for haberman’s survival dataset.

	Haberman’s Survival		
Algorithm	Normalization	Without Outliers	Without Outliers Normalized
**AdaBoostM1**	0.00	20.82	20.82
**BayesNet**	0.00	15.86	15.86
**BFTree**	0.10	21.06	21.42
**ComplementNaiveBayes**	N/A	16.73	N/A
**ConjunctiveRule**	0.00	16.55	16.55
**DecisionTable**	0.00	16.52	16.42
**DMNBtext**	−0.52	16.59	16.59
**IB1**	−0.19	27.93	27.93
**LWL**	0.00	19.10	19.10
**MultiBoostAB**	0.00	19.13	19.13
**MultiScheme**	0.00	16.59	16.59
**NaiveBayes**	−0.14	20.45	20.40
**NaiveBayesMultinomial**	N/A	17.29	N/A
**RandomForest**	−0.13	25.85	25.81
**RandomTree**	0.48	**28.37**	**28.29**
**RBFNetwork**	0.00	19.51	19.51
**SMO**	0.00	17.91	18.00
**Vote**	0.00	16.59	16.59
**VotedPerceptron**	**1.27**	16.57	19.81
**CHAT-OHM**	−3.91	24.08	24.17

**Table 10:** Comparison of classification improvement (%) for hepatitis disease dataset.

	Hepatitis Disease		
Algorithm	Normalization	Without Outliers	Without Outliers Normalized
**AdaBoostM1**	0.00	6.44	6.44
**BayesNet**	0.00	6.04	6.04
**BFTree**	−0.06	7.42	7.42
**CHAT-OHM**	4.56	3.98	4.22
**ComplementNaiveBayes**	N/A	9.18	N/A
**ConjunctiveRule**	0.06	6.26	7.34
**DecisionTable**	0.06	5.91	5.91
**DMNBtext**	**9.61**	6.65	**13.64**
**IB1**	0.00	6.68	6.68
**LWL**	0.25	6.01	6.01
**MultiBoostAB**	0.00	6.83	6.83
**MultiScheme**	0.00	6.65	6.65
**NaiveBayes**	−0.14	10.13	9.52
**NaiveBayesMultinomial**	N/A	9.72	N/A
**RandomForest**	−0.07	5.49	5.78
**RandomTree**	−1.62	8.33	6.35
**RBFNetwork**	0.00	**11.14**	11.14
**SMO**	−0.13	3.80	3.80
**Vote**	0.00	6.65	6.65
**VotedPerceptron**	6.94	7.31	8.88

**Table 11:** Comparison of classification improvement (%) for liver disorders dataset.

	Liver Disorders		
Algorithm	Normalization	Without Outliers	Without Outliers Normalized
**AdaBoostM1**	1.76	17.48	9.62
**BayesNet**	−0.23	**20.13**	9.95
**BFTree**	0.71	17.99	9.19
**CHAT-OHM**	−2.95	7.78	**12.68**
**ComplementNaiveBayes**	N/A	7.87	N/A
**ConjunctiveRule**	0.22	10.47	5.35
**DecisionTable**	−0.28	18.62	9.17
**DMNBtext**	**6.19**	8.23	11.30
**IB1**	1.03	12.91	6.97
**LWL**	−0.06	6.15	3.05
**MultiBoostAB**	−1.00	14.82	6.91
**MultiScheme**	0.00	8.23	4.12
**NaiveBayes**	0.53	3.65	1.96
**NaiveBayesMultinomial**	N/A	8.48	N/A
**RandomForest**	−0.68	18.92	8.82
**RandomTree**	−1.35	17.88	7.87
**RBFNetwork**	−0.25	13.97	6.86
**SMO**	−0.08	8.23	4.07
**Vote**	0.00	8.23	4.12
**VotedPerceptron**	2.33	10.36	8.57

For the Brest Cancer dataset, CHAT-OHM exhibits an improvement of 3.95%, being the second algorithm with higher improvement when removing the outliers. The dataset that presented greatest improvements with the removal of outliers was Haberman’s Survival. On average the classification accuracy improved from 71.82% to 91.49%. The improvements for this dataset vary from 15.86% to 28.37%. The CHAT-OHM exhibit an improvement of 24.08% when removing the outliers, positioning itself in the fourth place of the algorithms with higher improvements. For the Liver Disorders dataset the improvements when removing the outliers vary from 3.65% to 20.13%. The CHAT-OHM shows an improvement of 7.78%, which is relatively low when compared with the improvements presented by the rest of the algorithms for this dataset.

With the normalized outliers-free datasets, CHAT-OHM shows an improvement of 12.68% with the Liver Disorder dataset and its rank changes from the 9th place to the 4th place. Also, it was the classifier with the best improvement for this dataset. For the Haberman’s Survival dataset the model exhibit a 24.08% of increase in its performance and it was the fourth best improvement for this dataset.

## Conclusions

In this paper, we present a method that combines a Hybrid Associative classifier, a coding technique for output classes and a procedure of majority voting during the classification phase. This method is called CHAT-OHM. During the experimental phase, this method is applied to four different datasets related to the medical field. The performance of the method is compared with 19 machine learning algorithms implemented in WEKA Data Mining Software.

The proposed method uses an associative classifier, the CHAT [16], combined with a novel coding technique and a voting procedure. The results obtained demonstrate that the proposed method improved the result obtained by the CHAT.

However the experiments show that the CHAT-OHM is sensitive to the presence of outliers. To improve the classification accuracy of this algorithm, it has to be combined with a method of detection and removal of outliers. In the present work we use Wilson’s Edition as such method.

The CHAT-OHM presented fairly good results and a consistent behavior when applied to the four datasets used in this study. The performance of the model was not significant affected by the normalization process. On the other hand it was positive affected by the removal of outliers, displaying remarkable improvement in its performance, such as the ranking improvement for the Breast Cancer (4th place) with a performance increase of 3.95%. Another significant performance enhancement was obtained with the Liver Disorders dataset using normalization and outliers removal, the CHAT-OHM improved its rank to the 4th place with an increase of the classification accuracy of 12.68%.

It should be mentioned that our proposal is part of a family of methods based on the CHAT classifier. The main difference between these methods is the coding technique of each one, such as: Modified Johnson-Möbius binary coding, Gray coding, among others.

## References

[1] Steinbuch K (1961) Die lernmatrix. Kybernetik 1: 36–45.

[2] Willshaw DJ, Buneman OP, Longuet-Higgins HC (1969) Non-holographic associative memory. Nature 222: 960–962. 5789326 10.1038/222960a0

[3] Anderson JA (1972) A simple neural network generating an interactive memory. Mathematical Biosciences 14: 197–220.

[4] Kohonen T (1972) Correlation matrix memories. IEEE Transactions on Computers C-21: 353–359.

[5] Nakano K (1972) Associatron - a model of associative memory. IEEE Transactions on Systems, Man, and Cybernetics SMC-2: 380–388.

[6] Amari SI (1972) Pattern learning by self-organizing nets of threshold elements. System and Computing Controls 3: 15–22.

[7] Hopfield JJ (1982) Neural networks and physical systems with emergent collective computational abilities. Proceedings of the National Academy of Sciences 79: 2554–2558. 10.1073/pnas.79.8.2554 PMC346238 6953413

[8] Kosko B (1980) Bidirectional associative memories. IEEE Transactions on Systems, Man, and Cybernetics 18: 49–60.

[9] Ritter G, Sussner P, Diaz-de Leon J (1998) Morphological associative memories. IEEE Transactions on Neural Networks 9: 281–293. 18252452 10.1109/72.661123

[10] López-Yañez I, Argüelles-Cruz AJ, Camacho-Nieto O, Yañez-Marquez C (2011) Pollutants Time-Series Prediction Using the Gamma Classifier. International Journal of Computational Intelligence Systems 4: 680–711.

[11] Mathai G, Upadhyaya B (1989) Performance analysis and application of the bidirectional associative memory to industrial spectral signatures. International Joint Conference on Neural Networks 1: 33–37.

[12] Guzmán E, Pogrebnyak OB, Yáñez C, Moreno JA (2006) Image compression algorithm based on morphological associative memories. Progress in Pattern Recognition, Image Analysis and Applications 4225: 519–528.

[13] Chartier S, Lepage R (2002) Learning and extracting edges from images by a modified Hopfield neural network. Proceedings of the 16th International Conference on Pattern Recognition 3: 431–434.

[14] Yáñez-Márquez C, Diaz de León JL (2001). Lernmatrix de Steinbuch, Mexico: IT 48, Serie Verde, CIC-IPN.

[15] Yáñez-Márquez C, Diaz de León JL (2001). Linear Associator de Anderson-Kohonen, Mexico: IT 50, Serie Verde, CIC-IPN.

[16] Santiago-Montero R (2003). Clasificador Híbrido de Patrones basado en la Lernmatrix de Steinbuch y el Linear Associator de Anderson-Kohonen. MSc Thesis, CIC, IPN, Mexico.

[17] University of California, Irvine Machine Learning Repository website. Bache K, Lichman M (2013) UCI Machine Learning Repository. Available: http://archive.ics.uci.edu/ml Irvine, CA: University of California, School of Information and Computer Science. Accessed 2014 April 3.

[18] WEKA website. Hall M, Frank E, Holmes G, Pfahringer B, Reutemann P, Witten IH (2010) WEKA 3: Data mining software in java. Available: http://www.cs.waikato.ac.nz/ml/weka/Accessed 2014 April 3.

[19] Witten IH, Frank E, Hall M A (2011) Data Mining Practical Machine Learning Tools and Techniques. Elsevier.

[20] Breiman L (2001) Random forests. Machine Learning 45: 5–32.

[21] Platt JC (1998) Fast training of support vector machines using sequential minimal optimization. In: Schoelkopf B, Burges C, Smola A (Eds.). Advances in Kernel Methods–Support Vector Learning. MIT Press.

[22] Freund Y, Schapire RE (1996) Experiments with a new boosting algorithm. Thirteenth International Conference on Machine Learning: 148–156.

[23] Webb GI (2000) MultiBoosting: A technique for combining boosting and wagging. Machine Learning 40: 159–196.

[24] John GH, Langley P (1995) Estimating continuous distributions in Bayesian classifiers. Eleventh Conference on Uncertainty in Artificial Intelligence: 338–345.

[25] Christofides N (1975) Graph Theory: An Algorithmic Approach (Computer Science and Applied Mathematics). Orlando: Academic Press, Inc.

[26] Rennie JDM, Shih L, Teevan J, Karger DR (2003) Tackling the poor assumptions of Naïve Bayes text classifiers. Proceeding of the Twentieth International Conference on Machine Learning: 616–623.

[27] Kohavi R (1995) The power of decision tables. 8th European Conference on Machine Learning: 174–189.

[28] Wilson DL (1972) Asymptotic properties of nearest neighbor rule using edited data. IEEE Transactions on Systems, Man, and Cybernetics 3: 408–421.

[29] Kohavi R (1995) A study of cross-validation and bootstrap for accuracy estimation and model selection. Proceedings of the Fourteenth International Joint Conference on Artificial Intelligence 2: 1137–1145.

[30] Wolpert DH, Macready WG (1997) No free lunch theorems for optimization. IEEE Transactions on Evolutionary Computation 1: 67–82.

[31] Duda RO, Hart PE, Stork DG (2001) Pattern Classification. United State: Wiley.

